# Natural, Artificial, and Human Intelligences

**DOI:** 10.3390/jintelligence14100249

**Published:** 2026-10-08

**Authors:** Emmanuel M. Pothos, Dominic Widdows

**Affiliations:** 1 Department of Psychology, City St. George’s, University of London, London EC1V 0HB, UK; 2 Semantic Vectors Project, Bellevue, WA 98004, USA; dwiddows@gmail.com

**Keywords:** artificial intelligence, human intelligence, animal intelligence, chatbots, large language models, intelligence testing, Imitation Game, embodiment

## Abstract

Human achievement, whether in culture, science, or technology, is unparalleled in the known existence. This achievement is tied to the enormous communities of knowledge, made possible by language. We trace how the principle ‘᾿Eν ἀρχῇ ἦν ὁ λόγος’ (in the beginning was the word) was extended beyond its theological origins and heightened the value of written intelligence throughout Western societies. There lies the challenge regarding modern-age chatbots: they can ‘do’ language apparently as well as ourselves, and there is a natural question of whether they can be considered intelligent, in the same way as we are or otherwise. Are humans uniquely intelligent? We consider this question in terms of the psychological literature on intelligence, evidence for intelligence in non-human animals, the role of written language in science and technology, progress with artificial intelligence, the history of intelligence testing (for both humans and machines), and the role of embodiment in intelligence. We think that it is increasingly difficult to consider humans uniquely intelligent. There are current limitations in chatbots, e.g., concerning perceptual and social awareness, but much attention is currently devoted to overcoming such limitations.

## 1. Statement of the Problem: Are Chatbots Intelligent?

We are witnessing one of the most important and transformative technological revolutions in history, the emergence of artificial intelligence (AI). AI may be compared with inventions of historic significance, such as the printing press, steam engine, and the electronic computer—but compared with these, the economic and scientific impact of AI is being felt during a much more concentrated period of time. A discussion with a modern chatbot, such as chat GPT (we refer to large language models as chatbots in a generic way), is undeniably human-like and several studies have been establishing that chatbots display human cognitive traits, including similar biases (Binz & Schulz, 2023; Suri et al., 2024), the recognition of emotional states (Schaaff et al., 2023) and apparent empathy (Roshanaei et al., 2024), and other mental processing similarities (Mei et al., 2024; Strachan et al., 2024). One effect of this revolution is existential: we, humans, often assume our place in the world is unique by virtue of our intelligence, and so a conclusion of chatbot intelligence has the potential to change our conception of what it means to be human.

A practical question is whether chatbots will take our jobs. Together with being human-like, chatbots also encapsulate, in some sense, all human knowledge. Asking GPT-4 whether it will take my lecturer job, it is humble and reassuring: “As a university lecturer, you’re definitely in a role where expertise, creativity, and human connection are key. While AI, like ChatGPT, can help with things like answering questions, explaining concepts, or generating teaching materials, it’s not likely to replace the heart of your job.” However, increasingly in higher education, new policies are introduced to regulate the use of chatbots, given their dual ability to generate plausible coursework and to mark it with some competence (e.g., Gultom et al., 2024), questioning the role of humans in the process. Moreover, there are already indications that chatbots can carry out research roles (Lehr et al., 2024).

The observation that technological advancements can lead to job losses is hardly new—there is a name for this, “technological unemployment” (Peters, 2020)—and there are some compelling examples, e.g., the reduction of the U.S. workforce employed in agriculture between 1900 (41%) and 2000 (2%, Autor, 2014). Such concerns have been raised for chatbots too (Chace, 2016). The balancing perspective is that technological innovation also leads to new jobs (Autor, 2015; Mincer & Danninger, 2000) and over-productivity could be compensated in other ways (such as universal income). Additionally, some jobs require a human touch or do not depend on high levels of intelligence. What is unique about chatbots is that they are not ‘just’ another technological innovation with potential to eliminate some jobs: in other cases of technological automation, the replacement concerned tasks that were either too laborious (e.g., agricultural tasks) or seen as irrelevant to competencies which we have considered requiring intelligence (e.g., multitudes of rote arithmetical operations in accounting). Instead, with chatbots, we are faced with an artificial agent which seems just like us, concerning competencies tied to our notion of intelligence and so ones we have considered distinctly human.

A difficulty in addressing the question of chatbot intelligence is that our understanding of intelligence is itself incomplete. Some authors have attempted systematic frameworks for testing AI. For example, Laddaga and Robertson (2025) outlined several dimensions of intelligence, motivated mostly by psychological theory (Section 2). However, it is questionable whether these dimensions are necessary, jointly sufficient, or even independent. We contend that there is no definitive theory for understanding intelligence and, indeed, other related work acknowledges this limitation. For example, as in the present work, in E. K. Chen et al. (2026) and Holm and Banerjee (2025), there is also no attempt to define intelligence.

To make the present focus more concrete, the challenge is this: on the one hand, it appears that many competencies traditionally tied to intelligence can be covered by chatbots. On the other hand, it is possible that there is a special, unique ingredient in human intelligence, beyond these competencies, which underwrites human achievement. What is it about our minds which, for example, has enabled us to invent quantum mechanics?

In the absence of accepting a working definition of intelligence, we consider different sources of theory and evidence, including psychological theories of human intelligence (can they help us understand what, if anything, is unique about human intelligence? Section 2) and animal intelligence (do comparisons with animals help in this discussion? Section 3). Failing to come up with some concrete insight from Section 2 and Section 3, we then take a step back and try to draw inspiration from a brief historical overview of human civilisation. This helps establish the catalysing role of language and so brings into focus the significance of having these linguistically capable artificial agents today. This brief historical overview also helps explain why language and symbolic thought generally (e.g., mathematics) have ended up acquiring such enormous importance in our notion of human intelligence and our approaches to intelligence testing. We follow with a brief overview of the historical origins of AI (Section 5). Section 6 addresses the question of testing for intelligence—we will see that modern chatbots display excellent performance on all existing tests. Section 7 considers whether the over-emphasis on language and symbol-manipulation (regarding both intelligence theories and testing) might create blind spots in this discussion; in that section we briefly review embodiment. In Section 8 we summarise our conclusion that chatbots should be considered, for most practical purposes, intelligent and address common objections to such a conclusion.

Similar questions have attracted much attention. E. K. Chen et al. (2026) conclude that artificial general intelligence is already here. They review a set of common objections (some of which we also consider below), dismissing all of them. Our approach, instead, is to focus on whether there might be some unique ingredient in human intelligence. Holm and Banerjee (2025) conclude that “AI is neither artificial nor intelligent.” Also, Gignac and Szodorai (2024) evaluate chatbot intelligence more as expertise and achievement, rather than general intelligence. Our discussion points us to an opposite conclusion.

## 2. What Is Intelligence: Psychological Approaches

Attempts to define intelligence long predate modern psychology. In Western philosophy, intelligence has been traditionally connected with reason and rational thought (Gardner, 2011, Chapter 1). Aristotle associates reasoning particularly with the concept of λογος (which literally means speech, though it can also mean logic), a historic connection discussed in Section 4.

For Descartes, awareness of one’s own thoughts proved existence itself (cogito ergo sum). This tradition takes on a modern form in the work of Freud and Jung, who made introspection about the self and its symbolic life central to the emerging science of psychology.

There has also long been a desire for more empirical foundations—Hume, for example, was particularly sceptical of self-reported self-awareness as a scientific grounding. Mathematicians including Leibniz and Boole proposed ways to formalize and even automate reasoning, and Whitehead extended such symbolic approaches into broader theories of abstraction and meaning. These efforts eventually aligned intelligence with symbolic operations that could be both economically advantageous and systematically measured, which we will see became common practice in the later 1800s.

In psychology, by the late 1800s, figures such as Galton, Wundt, and William James had begun to turn these ambitions into methods, using reaction times, association tests, and other measures to make mental processes experimentally observable. By this point, Western psychology largely treated intelligence as a property of individual minds, though with different approaches to studying those minds. We highlight below some of the main ideas (for a more comprehensive review readers should refer to e.g., Laddaga & Robertson, 2025).

As certain kinds of intelligence could be tested, it became tempting to define intelligence in terms of what such tests could measure. Much of the psychological tradition regarding intelligence concerns psychometric theories, which aim to explain individual differences in test scores, on the basis of factors (e.g., see Sternberg, 2012). The earliest proposal of this kind is due to Spearman (1904), who argued for two factors, a general one (later associated with ‘mental energy’) and a specific one, comprised of competencies for particular tasks. Thurstone (1938), instead, identified seven “primary mental abilities” as the determinants of intelligence. These mental abilities include verbal fluency, spatial visualization, and perceptual speed. While originally Spearman (1904) and Thurstone (1938) were interpreted as antithetical, this is not necessarily the case. One way to reconcile the two views is if the general vs. more specific intelligence factors are hierarchical, so that the latter informs specific aspects of the former. This hierarchical approach has been taken up by other intelligence psychologists, such as Carroll (1993), who expanded both the specific and general abilities.

This literature has recognised that some abilities reflect knowledge, while others reflect processing power (in some general sense). Cattell (1943) introduced the distinction between fluid and crystallized abilities, with the former concerning performance that depends minimally on prior knowledge (e.g., problem-solving on a novel task) and conversely for the latter (e.g., vocabulary tasks). There is a converse relation between these two kinds of abilities and age, with fluid decreasing, while crystallized increasing with age (e.g., Ghisletta et al., 2012).

A more process account of intelligence has been that intelligence reflects the speed of information processing (Deary et al., 2001), sometimes in simple perceptual tasks, such as whether two lines are the same length (MacKintosh, 1981, but see Kiessling & Hentschel, 1988). There have been related proposals. For example, Hunt et al. (1973) considered the speed with which information is ’absorbed’ into memory and subsequently retrieved. Such approaches do not depend on the extent of knowledge or other culture-specific skills, such as social skills.

There have been other attempts to understand intelligence in more cognitive terms, e.g., in terms of working memory capacity (Conway & Engle, 1996), underpinned by high correlations between measures of working memory and reasoning ability (e.g., Kyllonen & Christal, 1990; Ackerman et al., 2005). Interestingly, in cognitive science, working memory capacity limitations have been argued to enable learning in complex domains (Newport, 1988).

Another processing idea is that intelligence relates to the ability to solve problems and achieve goals. Sternberg (1985) proposed that intelligence is characterized by three abilities: practical intelligence (environment interaction), creative intelligence (new problems and situations), and analytical intelligence (primarily problem-solving). Problem-solving ability has had a prominent role in other proposals (e.g., Danner et al., 2011; Hambrick et al., 2019), as well as often being a component in many others (starting from Spearman, 1904). Sternberg’s (1985) theory is an example of approaches positing multiple types of intelligence. Gardner (2006) proposed a longer list, including musical, interpersonal, spatial, and linguistic intelligence, and Salovey and Mayer (1990) introduced the idea of emotional intelligence as the ability to perceive and utilize emotions in cognition.

Psychometric approaches to intelligence testing have demonstrated much practical value. For example, they predict longevity (Deary, 2001; Deary et al., 2007), health outcomes (Hart et al., 2003), job performance (Schmidt & Hunter, 2004; Ericsson & Pool, 2016), and academic success (Deary et al., 2007). Particular aspects of intelligence have been prioritised in intelligence testing: the approaches of Spearman (1904) and Binet and Simon (1905) were translated into educational and cultural settings that emphasized the importance of written answers to prearranged test questions (Rogele, 2020). A preference for written demonstrations of intelligence, particularly in English-speaking educational systems, has profoundly affected the way AI has been viewed and pursued, a relationship that we explore throughout the rest of this paper.

One might wonder whether developmental theories of intelligence might offer an alternative perspective. There is a rich and extensive corresponding literature. Such theories examine how cognitive capacities emerge and change across the lifespan; they provide an important account of the origins of human abilities. However, our present question is comparative rather than developmental: whether the capacities commonly associated with intelligence are uniquely human, rather than how those capacities are acquired in humans. Developmental accounts therefore do not substantially alter our conclusions in this section.

Summing up, this is a complex and rich literature, and we only want to touch upon a handful of points, as relevant to this discussion. Can the discussion of psychometric theories of intelligence inform the question of chatbot intelligence? Broadly speaking, psychometric theories have sought to understand human intelligence in two ways. First, there are accounts concerning processing characteristics. If the critical characteristic of human intelligence is related to processing, then we can readily conclude that chatbots can easily attain human intelligence. Chatbots already exceed human intelligence in terms of speed, memory capacity, information absorption, etc. Second, there are theories of human intelligence concerning different facets, such as spatial, emotional, musical, etc. (cf. Gardner, 2006). So, regarding chatbot intelligence, we can pose the question: are there facets thatcannot be covered by chatbots? Note that this question is somewhat ill-formed, because there is no definitive account of the relationships and relative importance between the various proposed facets of human intelligence (cf. Corr & Krupic, 2024). In any case, looking at the various proposals for the facets of human intelligence, the conclusion would be that most of these are already reflected in chatbot performance (linguistic generally; problem solving; reasoning etc.; see also Section 6). There are some facets for which currently there is less evidence for chatbots, such as emotional and social (the issue of embodied intelligence is considered in Section 6). Still, we contend that the theories briefly considered in this section do not reveal aspects of human intelligence not currently covered or insurmountable for chatbots.

## 3. Animal Intelligence

The brief consideration of theories of human intelligence did not reveal any clear insights that can guide the question of human vs. chatbot intelligence. The question of whether human intelligence is unique or not also arises in relation to animal cognition, and perhaps the relevant insights might guide reflection on chatbot intelligence as well.

Humans are not the fastest, toughest, most enduring, etc., creatures, but we are distinguished for our cultural, technological, and scientific accomplishments—our achievement in these respects surpasses that of all other creatures. This has allowed us to consider ourselves the most intelligent species. Even if this is true in some sense, there is a question of whether other species are intelligent like us, but to a lesser extent, less intelligent in fundamental ways, or perhaps in some ways more intelligent than humans have realised. Therefore, we briefly consider comparative discussions between human and animal intelligence and so hope to draw inspiration regarding chatbots.

There are signs of intelligence throughout the animal kingdom (Burkart et al., 2017). For example, dogs have highly developed social awareness, including sensitivity to human body language, human gestures, and parts of language (Hare et al., 2002; Kaminski et al., 2004). Dogs are able to solve a variety of logical/reasoning problems, such as a means-end task (Range et al., 2011) or spatial puzzles (Barrera et al., 2015). Theory of mind is the ability to attribute mental states (such as beliefs and desires) to others. It is typically considered an integral part of intelligence, especially social intelligence (Navarro, 2022). There have been arguments that dogs and other primates have a theory of mind (Catala et al., 2017; Premack & Woodruff, 1978), though there is controversy (Heyes, 1998). Chimpanzees have extensive tool-using abilities, including for food gathering/hunting, grooming, and communication (Koops et al., 2015). Chimpanzees can recognize abstract symbols (Rumbaugh & Washburn, 2003) and can innovate solutions to unfamiliar problems (Bandini & Harrison, 2020). Similar evidence has accumulated for other species, e.g., corvids, who appear to have problem-solving abilities comparable to those of chimpanzees (Güntürkün et al., 2024). Perhaps unsurprisingly, there is much evidence regarding musical intelligence in birds, e.g., relating to rhythm perception and processing (Fitch, 2015; Patel, 2006) or the structural properties of birdsong (Kawaji et al., 2024; Y. Zhang et al., 2023). Further, there is some evidence of musical synchronisation amongst birds, though for duetting birds at most (Hall, 2009; Mann et al., 2005). Overall, bird musical ability does not appear to match the complexity of human music (Lerdahl & Jackendoff, 1983; Levitin, 2006).

What about, for example, the homing instinct of a pigeon or the way a leopard calculates the exact time and distance before it pounces on prey? For example, pigeons have been known to successfully return home across distances exceeding 100 km (Schmidt-Koenig & Schlichte, 1972). We would not call an individual pigeon intelligent for demonstrating its homing instinct, in the same way we would not call an individual human intelligent for learning to walk or ride a bicycle—such skills are considered default competencies that most members of a species are endowed with, as a result of evolutionary adaptation. Such competencies seem better considered as intelligence at the species level, allowing for different competencies developed through evolution (e.g., Jerison & Jerison, 1988). A similar case can be made regarding the remarkable ingenuity in, for example, the way ants or bees build their nests (Winston, 1987).

Such examples show that evolutionary intelligence and the intelligence of individual agents are distinct. Often the two types of intelligence are related. For example, a child’s ability to correctly generalise from few linguistic exemplars (supporting language learning and competence, which is an aspect of an individual’s intelligence) plausibly reflects structures which have been developed in the human brain across many generations. That is, it is thought that an individual person’s efficiency and speed of language learning is supported by innate information (e.g., Jerison & Jerison, 1988).

Following from the example just above, for humans at least, linguistic competence seems to be partly an instinct (e.g., Pinker, 2015). Modern chatbots can become linguistically competent, but only after much training—they lack a putative language instinct. That is, linguistic capacity in humans reflects the combination of intelligence of individual agents and evolutionary intelligence/adaptation, whereas for chatbots only the former applies. We think similar arguments can be made regarding many competencies in humans or animals. As an aside, regarding language, we think the crucial point for intelligence is less so linguistic competence as such, but rather the fact that complex thought is typically (not always) communicated linguistically. Are modern chatbots also capable of complex thought to the extent that we can consider them intelligent?

Self-interest is evident throughout animal behaviour (for example, taking steps to avoid being eaten). Self-awareness has also been studied in animals, including the famous finding of Gallup (1970) that chimpanzees, but not monkeys, learn to recognize a reflection in a mirror as their own. It is tempting to assume that humans are uniquely self-aware, hence more intelligent than other animals, but attempts to describe or demonstrate something distinct regarding self-awareness in humans have been empirically challenging (Carruthers, 2009). Regarding chatbots, there is certainly some evidence that they can self-monitor, though self-awareness is a different matter (Section 6).

Beyond evolutionary intelligence, for some species, there is collective intelligence, the ability to achieve more than what an individual could accomplish, through cooperation. In the animal kingdom, for example, ants use pheromones to communicate about food sources or dangers and often work together in foraging tasks (Hölldobler & Wilson, 1990); or the way African wild dogs coordinate in hunting has sophisticated characteristics, including role differentiation and vocal communication (Creel & Creel, 2002; for an example regarding rats see Schuster, 2002). However, no other species has been more successful in transcending what is possible for an individual than humans. Fernbach and Sloman (2018) coined the term ‘community of knowledge’ for the idea that even seemingly simple aspects of our lives are underwritten by massive knowledge contributions of which we are mostly ignorant. Communities of knowledge describe communities of individuals who collectively benefit from knowledge across the community, even if many members are unaware of this knowledge. Our ability to form sophisticated communities of knowledge is fundamentally linked to the unique expressivity of human language (e.g., Lakoff & Johnson, 2003; Pinker, 2015) and the fact that our language has a written form (Latour & Woolgar, 1987). Written language has enabled global communities of knowledge; arguably, it is the cornerstone of our civilization (Goody, 1977; e.g., see Gaudet, 2014, for an interesting proposal concerning the interaction between climate, the durability of early writing mediums, such as papyrus, and the emergence of early civilizations).

Is there any evidence for art, science, technology amongst non-humans? Regarding art, some animals can learn to discriminate based on the aesthetics of pictures and show preference towards higher aesthetics (e.g., songbirds, for musical stimuli; Watanabe, 2013). Some birds create and decorate elaborate structures to attract mates (Borgia, 1985), and some animals can create pictorial patterns in the sand or using rudimentary tools, which resemble artistic expression (Pelé et al., 2021). However, such evidence is a far cry from demonstrations that non-human animals show human-like appreciation of these behaviours and their outputs, in the way we do for art (English et al., 2014). Regarding science, the scientific endeavour implies experimentation and exploration of testable hypotheses to identify general principles and so generalize from known situations to novel ones. There is no evidence that animals can do science. However, there is some evidence that animals can experiment and adjust strategies, create artifacts, and develop rudimentary tools. For example, chimpanzees make tools to help them forage for ants or termites, through a process of successive improvement before the desired form is achieved, sometimes before actual tool use (indicating that they have an idea of the required form for a tool prior to use; Boesch & Boesch, 1990). New Caledonian crows have been reported to construct hooked tools to aid them in insect capture (Hunt et al., 1973). Moreover, there is evidence of animals adapting behaviour from trial and error and changing circumstances (e.g., Fiorito & Scotto, 1992).

Such evidence indicates that animals are capable of technology and innovation, though it appears that their only path is through gradual trial and error, as opposed to the sudden realization which often accompanies human technological innovation (famous examples are penicillin and the discovery of the chemical structure of benzene (Fleming, 1929; Kekulé, 1865, as cited in Rocke, 2015).

There are two conclusions we wish to draw from this discussion. First, although it might look like there is something special about human intelligence compared to animal intelligence, it is hard to identify what this is. Second, whatever else, a huge factor in what can broadly be considered human civilisation is language. To appreciate the extent of the impact of having language and implications regarding human vs. chatbot intelligence, we next turn to a brief historical overview.

## 4. The Word and Western Culture

Electronic computing and AI are recent products, driven by relationships between technology and science, that arose along with industrialization, in a culture that particularly prized written forms of intelligence. This section summarizes aspects of these cultural developments and how they influenced some of the development and attitudes towards artificial intelligence.

In standard historical accounts, Western civilization is the product of Hebrew religion and Greek science, becoming the Judeo-Christian tradition. Both of these roots embody the distinctiveness of human origins and intelligence, with an emphasis on knowledge expressed in words. In Genesis (Ch 3), Adam and Eve eat the fruit of “the tree of the knowledge of good and evil” and ostensibly become self-aware. In Aristotle’s Politics (Bk VII Ch 3), “Virtue arises through nature (physis), habit (ethos), and reason (logos)”, and while some animals demonstrate ethos as well as physis, only humans have logos. This combination became central to Christianity, whose most mystical Gospel starts with “In the beginning was the Word” (᾿Eν ἀρχῇ ἦν ὁ λόγος, John Ch 1).

This did not originally imply that knowledge and intelligence are expressed in written words. Many great scholarly traditions have emphasized oral learning, and many scriptural and epic works were known and taught long before they were written down in canonical manuscripts. Plato’s Phaedrus dialogue raises fears that those who rely on writing “will not use their memories … they will appear to be omniscient and will generally know nothing,” which is analogous to arguments today that artificial language technologies reduce human skills.

**Figure 1:**
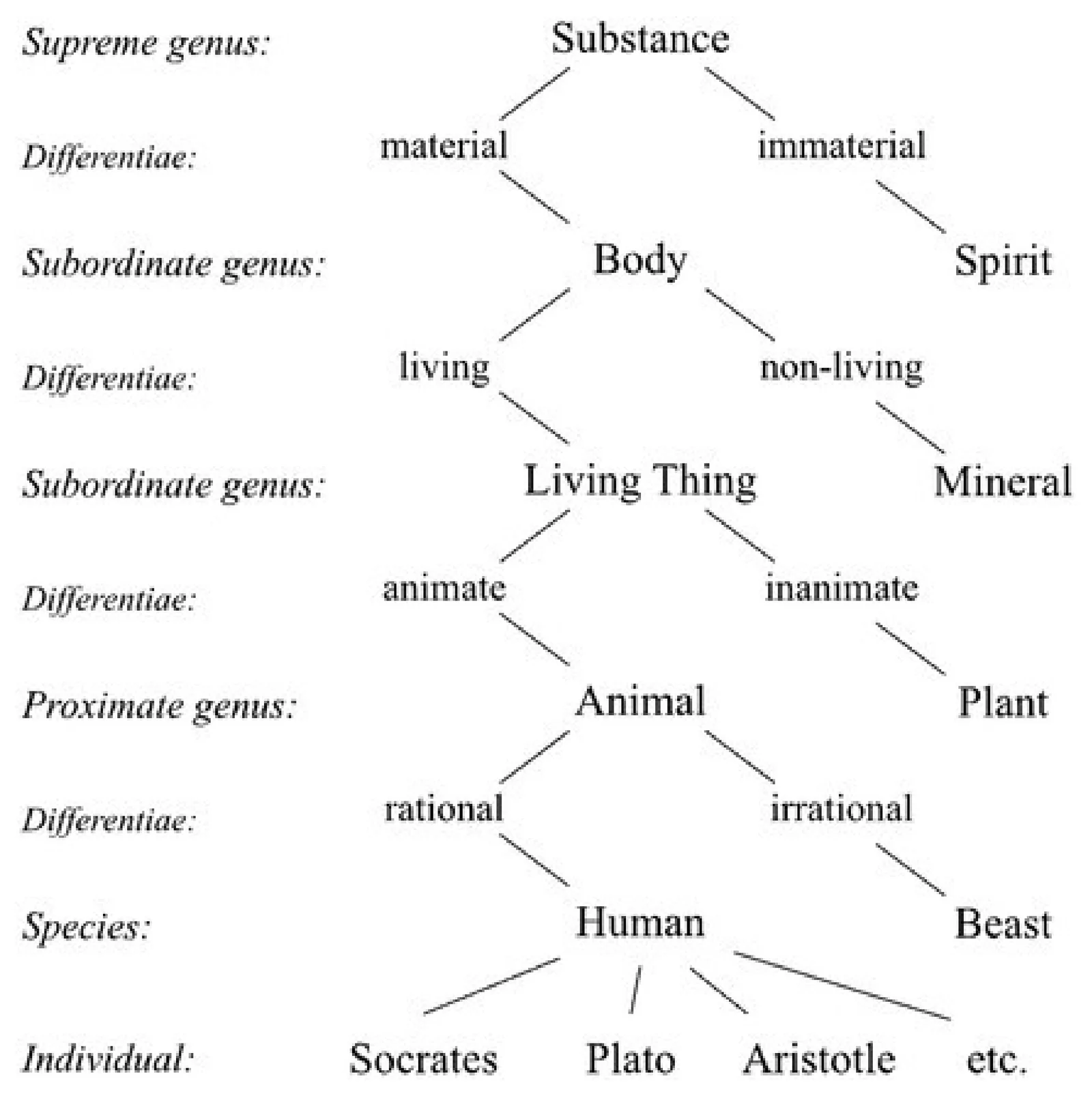
Tree of Porphyry, used to teach introductory logic from the 3rd to the 19th centuries in European schools.

As Christianity became established, so did the belief that humans are verbal, rational animals, and that this is the divine core of our identity. Beyond religious education, this was taught in introductory logic and classification (we use classification in the logical sense) from the third century onwards, using the tree of Porphyry (Figure 1), perhaps history’s most famous decision-tree. It was used until the mid-1800s, when Boolean propositional calculus and truth-tables supplanted Aristotelian classification and syllogism in the teaching of logic (Boole, 1854/1958). The claim that humans are uniquely rational was not just part of biology and theology; it was taught as a foundational principle in Western logic.

The primacy of written words was established gradually, and not only in Europe. The Qur’an emphasizes the sacred status of its own words and respect for Jewish and Christian scriptures, whose followers are described as “people of the Book” (Surah 5:65–69). In China, the tradition of Confucian literary scholarship became central to the famous civil service examinations and by the Ming period (1368–1644) had become the main route to official status. In theory, this was a great source of social mobility, and historically the first great example of ‘standardized testing’ (Elman, 2013).

North-western Europe came to rely especially on writing as a source of truth and intelligence. The widespread use of printing, and the Protestant Reformation, emphasized the view that the Bible itself was literally the Word of God, rather than the more mystical ‘Word made Flesh’. In the Renaissance period, famous scholars were often multi-disciplinary, and their works included sculpture, art, poetry and music; but for scholars of the later Enlightenment period, by ‘collected works’, we tend to mean a book with just writings. In music, a Western European tradition developed in which composers are celebrated, but by their ‘works’ we mean written manuscripts. In Western classical ensembles, the paper with the written notation is even called the music, whereas in jazz ensembles, the paper documents are called charts.

The independence of science itself from roots in natural philosophy and common engineering practices also evolved during this era. In the 1600s, the slide rule (based on Napier’s logarithms) and the pendulum clock (based on Galileo’s mechanics) were the first examples of theoretical discoveries, described in writing, leading directly to the creation of new devices.

Steam engines became pivotal in the Industrial Revolution and illustrate the emergence of a new, causal relationship between technology and science. Ancient inventors, including Heron of Alexandria, had built demonstration steam engines, but they were never used to replace human labour. From the early 1700s, Newcomen’s steam engines were in active use pumping water out of mines, but the steam was heated and then condensed in the same chamber, an expensive and dangerous combination. Watt’s brilliant innovation during the 1760s was to separate these duties between two different chambers, leading to reliable rotational power that could drain mines, then power a whole weaving factory, and eventually leading to steamships and locomotives. In trying to understand the relationship between heat inside boilers and mechanical work in piston chambers, scientists including Carnot, Kelvin, and Clausius developed modern thermodynamics between the 1820s and 1860s. Thermodynamics was arguably the first branch of physics that was deliberately motivated by the goal of optimizing an existing technology.

Maxwell’s electromagnetic theory in the 1860s went leaps further, explaining the propagation of light waves in traditional optics and predicting the behaviour of an entire spectrum of oscillations, invisible to human senses. During the 1880s, Hertz used these theoretical predictions to transmit and receive real radio waves. This formed a new relationship: science had brought about a technology that was beyond our natural imagination (Oerter, 2006, Chapter 1). In this new relationship, written works of science led directly to new kinds of machines, and scientific progress enabled a landslide of innovations throughout the late 1800s and early 1900s.

Since that time, it has often been assumed that written science is the main source of technological inspiration and reliability. Electronic computing is a clear example of technology that arose as the brainchild of written science and has inherited some of these presuppositions (with many very successful results). Nonetheless, a small sample of inventions (fermentation, wheels, metalworking, textiles, clocks, gunpowder, printing, telescopes, steam engines, dried noodles, vacuum cleaners, velcro) demonstrates immediately that written science is just one path to invention (Mokyr, 1992).

Anglo-American educational practices have particularly emphasized written assessments. While the French Baccalaureate and German Abitur exams, formalized since the early 1800s, included practical and oral components, British qualifications, like the Chinese civil exams long before, were standardized entirely by writing. Given the emphasis on STEM education today, it is easy to assume that science education was developed along with science itself, but this would be an anachronism: Newton, Priestley, Cavendish, and Darwin did not go to secondary schools with classes in physics, chemistry, and biology, but instead, aside from geometry and algebra, a principal focus would have been classical languages and literature. British “grammar schools” had existed since the late Middle Ages—the redirection of endowment funds for “other useful Branches of Literature and Science in addition to or (subject to the Provisions hereinafter contained) in lieu of the Greek and Latin Languages” was controversial enough to motivate the Grammar Schools Act of 1840 (Parliament of the United Kingdom, 1840). The use of written examinations as the measure of intelligence was explicitly promoted in the Northcote and Trevelyan report that led to the British Civil Service exams. Their main stated goals included promoting merit over patronage, and also “establishing a proper distinction between mechanical and intellectual labour” (Northcote & Trevelyan, 1854). While moral and physical fitness was to be vouched for in person, for the purposes of choosing civil servants, intellectual labour was identified with writing. Overall, such tests prioritized written over manual skills: to fix a broken boiler, the person who could write down the problem, order the replacement parts, etc., was considered intelligent, whereas the person who actually mended the boiler less so.

Well before the time the term AI was coined, assumptions about the relationship between science, schooling, written words, and intelligence were part of the fabric of Western European culture. For centuries, there were serious philosophical and scientific claims that abstract reasoning is impossible without language, and only recently have brain imaging studies shown that these activities are dissociated from one another (Fedorenko et al., 2024).

Language and reasoning abilities have clearly helped to distinguish us from other species, and writing as a technology has worked as a great catalyst for improving, and even helping invent, other technologies. Not surprisingly, the most successful industrial societies have prized and cultivated writing skills. These long-cherished priorities have made its beneficiaries clearly separate from beasts, but particularly susceptible to the challenging shock of fluent chatbots. Turing even anticipated such cultural resistance, saying “It is likely to be quite strong in intellectual people, since they value the power of thinking more highly than others (Turing, 1950, §6.II)”.

This overemphasis on language/symbol-based kinds of intelligence has two further implications. First, it has been distorting our understanding of what intelligence is, when there are many types of intelligence not tied to language/symbol manipulation. This is not to say that being intelligent in language is not significant, and we agree that “…chatbots’ linguistic mastery is genuinely significant for intelligence comparisons” (as a reviewer of this paper noted). Second, because of the facilitating effect of language through communities of knowledge, we think that we (humans) tend to overestimate our intelligence at the individual level.

A critic can certainly argue that the above narrative, while indicative, certainly does not constitute a causal demonstration between language, our (humans’) impression of intelligence, and the development of human civilisation. Nevertheless, the purpose in this section is not to attempt a demonstration of corresponding causal connections—rather, to provide our (the authors’) opinion of how linguistic mastery has ended up being so central in these debates.

## 5. Artificial Intelligence Joins the Stage

The brief historical overview in the previous section naturally leads us to consider the historical origins of AI. Indeed, the first electronic computers are a great example of a scientific discovery, based on written work, leading directly to new technologies. The theory of computing was developed by Church and Turing in the 1930s, and Turing’s calculating machines were built in the hidden limelight of wartime codebreaking in the 1940s. By the end of the decade, Turing, von Neumann, and many others had developed reprogrammable machines, whose instructions and calculations were stored in electronic memory and were capable of performing thousands of arithmetic calculations within seconds, using cascading patterns of Boolean logic to process numbers represented in binary digits.

Shannon’s landmark Mathematical Theory of Communication (C. Shannon, 1948) developed the quantification of information, using the statistics of character and word sequences in English as grounding examples. These are still foundational examples in statistical natural language processing: Shannon’s zeroth-, first-, and second-order approximations to English would nowadays be called n-gram models (Manning & Schütze, 1999, Chapter 6). Rather than trying to program and run these models on an early electronic computer, Shannon’s samples were taken from random pages in books—but he still accurately described the computational possibilities, and scaling limitations, of n-gram models.

Turing’s paper on Computing Machinery and Intelligence in 1950 (Turing, 1950) is a foundation of the field that was eventually called AI. Turing’s proposal was particularly important for text processing, introducing a challenge called the Imitation Game. Two humans, or a human and a machine, try to imitate one another, communicating directly with a judge, with interactions restricted to the inputs and outputs of a teleprinter. Whether the judge can distinguish a machine from a human through this interface alone has come to be called the Turing Test. Turing carefully avoided the question of whether such a machine is ’thinking’, saying instead that such behaviour would typically be regarded as demonstrating intelligence. His paper examined common objections to the idea that such a machine could be called intelligent (e.g., not having a soul or just regurgitating things it had across before), arguing that they are rooted in prejudice and other mistakes, and speculated that the most effective way to build such a machine would be to create a ‘child machine’ with the programming and resources to be able to learn, and provide this machine with training experiences. He estimated that such a machine could be built by the year 2000 and, while that timeline was not achieved, Turing’s paper broadly anticipated the current approach to language processing, and the tendencies to resist these achievements.

Some of the core components of today’s chatbots were pioneered in the 1950s and 1960s. Most famously, Rosenblatt’s invention of the perceptron (Rosenblatt, 1958) and Widrow and Hoff’s use of continuous, gradient-based iterations to train perceptron networks (Widrow & Hoff, 1960) are generally considered the precursors of today’s neural networks (Raschka et al., 2022, Chapter 2). Representing words as vectors learned from data (usually called ‘word embeddings’ since the success of the word3vec package, Mikolov et al. (2013)) was pioneered in the SMART (System for the Mechanical Analysis and Retrieval of Text) project, led by Gerard Salton during the 1960s (Salton, 1971). The success of the SMART system was demonstrated using early statistical evaluations of precision and recall, developed during the Cranfield experiments, whose purpose was indexing and retrieval techniques, particularly for scientific reference and military intelligence gathering (Cleverdon et al., 1966). The Cranfield project was foundational in information retrieval, not only for its results, but for establishing the practice of evaluating search engines by measuring the precision and recall of sets of search results, when compared with held-out test data. This practice was followed in the 1990s in the Text Retrieval Evaluation Conference series (Harman, 1993), and submitting results to a “TREC Style Evaluation” is a key forerunner of many machine learning metrics and leaderboards, such as Kaggle and the MLCommons, used to compare models today.

One of the key surprises from the Cranfield experiments was that automated indexing strategies, based purely on the words occurring in each document, outperformed indexing by human experts. This surprised, and initially dismayed researchers: “the results which seem to offend against every canon on which we were trained as librarians” (Cleverdon et al., 1966). ‘Computer’ had been a job title for skilled arithmeticians during World War II, but since the rules of arithmetic had been formalized, it stood to reason that a faster electronic machine that performed arithmetic could produce results that were just as good. ‘Indexer’, however, was a different matter: the job had been performed by expert librarians since the 1800s, and the notion that skilled work with words could be done by a machine just as well, if not better, was a shock. Statistical term-weighting (i.e., how much weight should a term be given in a retrieval engine) is an example: instead of weighting terms using controlled vocabularies of keywords and subject headings, it worked better to assign weights, or importance scores, to each word in a document, based on how many times that word occurs in that document and how few other documents it occurs in. This family of weighting schemes is called tf-idf (term frequency, inverse document frequency). The insight that statistical term-weighting (or term specificity) is modelled so effectively by tf-idf is due particularly to Spärck Jones (1972), and though it might be considered a blunt challenge to more formal logical semantics, it is today common to trace the linguistic and philosophical heritage of such statistical observations to the works of Firth (1957) and Wittgenstein (1953/2001).

Given such strong foundations, it may be surprising that statistical language processing using neural networks took so long to fully flourish. One reason is that it required decades of hardware advancements, including the development of graphics processing units and their adaptation to neural networks, to realize the potential of neural network methods. Another factor is that most research in AI and computational linguistics during the later decades of the 1900s was focused elsewhere, particularly on symbolic, logical, and syntactic methods. In linguistics, Chomsky wielded great influence, and his early work on syntactic structures claimed both that “The most that can be reasonably expected of linguistic theory is that it shall provide an evaluation procedure for grammars” and “probabilistic models give no particular insight into some of the basic problems of syntactic structure” (Chomsky, 1957). In AI, the book Perceptrons: An Introduction to Computational Geometry (Minsky & Papert, 1969/2017) demonstrated key problems encountered by neural networks with the learning of non-additive functions, including cases as simple as the logical XOR. As key inventors of early neural networks, Minsky and Papert emphasized in their book that their objections were not meant to divide the field, but rather that research on neural networks would fade as other ideas took over; the 1988 edition included an epilogue saying, “In any case, the 1970s became the golden age of a new field of research into the representation of knowledge.” The new techniques listed as part of this ’golden age’ included word-expert parsers, nonmonotonic logic, and planning procedures, which led many researchers to “set aside their interest in the study of learning in favour of examining the representation of knowledge.” With hindsight, this is seen more as a dead-end than a golden age and the beginning of a period that is sometimes called the “AI winter.” (Ironically, the term AI winter was also coined by Minsky, during the 1980s, as a warning against what might happen if expert systems did not bear fruit.) But for some decades, symbolic logic was considered to be the core of artificial intelligence and linguistic theory (Spärck Jones, 1994), and the most established textbooks on mathematical and computational linguistics from this era focus on discrete symbolisms, barely mentioning statistics, probability, or continuous mathematical foundations, such as vectors and gradients (Partee et al., 1993; Allen, 1995). Overall, symbolic AI work has led to many contributions from these endeavours—just one of many examples of active areas of research concerns multi-modal entity alignment in relation to record-linkage and knowledge-graph integration, e.g., Z. Chen et al. (2023). Still, it has only been with the advent of modern chatbots that serious discussions about machine intelligence at human levels have begun.

Today’s chatbots come from the very techniques that were supposed to have been left behind: machine learning in general and neural networks in particular are the cornerstones of AI today. During the 1980s, researchers including Hinton and LeCun developed the backpropagation method, which enables deep neural networks to be trained efficiently by passing feedback on classification results back through layers in the network, using the chain rule from multivariable calculus (Rumelhart et al., 1986). Backpropagation was popularized by Rumelhart et al. (1986), who noted that related variants had been discovered independently by David Parker and Yann LeCun. LeCun (1985) had already introduced a multilayer supervised learning procedure using backward propagation of target information through hidden layers, i.e., an early form of backpropagation. Neural nets built in this way began to outperform competing approaches on several pattern-recognition tasks, including handwritten-digit recognition (LeCun & Bengio, 1998). In 1997, Deep Blue, a chess computer program invented by Hsu, a doctoral student at Carnegie Mellon University, defeated the world chess champion Gary Kasparov, relying on a massively parallel expert fuzzy-rule-based system and effective use of a dataset of Grandmaster games (Campbell et al., 2002). In the 2000s, the first neural net model for next-word prediction was trained successfully to produce relatively consistent natural language phrases, over longer context distances than those achieved with n-gram models. AI was beginning to produce systems that outperformed humans on complex symbolic tasks and, rather than doing this using rules created by human domain experts, this was achieved by leveraging fast, massive computation. The game of Go, more combinatorically demanding than chess by a wide margin, was mastered by the Alpha Go system, using databases of real games for training and then, even more strikingly, by Alpha Go Zero, which learned solely by playing against itself, with no human supervision except for being given the rules and the resources to simulate play (Silver et al., 2017).

Early on, attempts at AI development focused on creating systems which would excel at specific tasks, assuming that such systems put together would create a human-like, general intelligence: for example, Shannon famously proposed that the game of chess would be an ideal challenge for developing intelligent systems, since it is generally considered to require “thinking” for skilful play (C. E. Shannon, 1950). Based on the combinatoric explosion of possible chess positions, Shannon argued that a brute-force approach, leveraging large computational power, would at best enable the creation of a weak chess player. But fast forward a few decades, and it turns out that one can design an extremely effective chess system, mainly by applying brute-force computation over large datasets. This is both an insight and a challenge to humans; some of the most poignant reflections on this theme have been written by Kasparov himself (Kasparov, 2017).

AI agents have become better by carefully leveraging larger example datasets and computational resources, a process which contrasts with the way humans accomplish similar expertise, typically requiring years of devotion Gladwell (2008). Understandably, this is often unwelcome news to human experts, and the theme that general methods that leverage computation are ultimately the most effective, and by a large margin, has even been dubbed “The Bitter Lesson” (Sutton, 2019). But the two questions—are we trying to construct systems which behave intelligently, and are we trying to re-create human intelligence—are not the same, though the recognition of this difference has taken some time (Russell & Norvig, 2022, Chapter 1).

The large language models (LLMs) that drive modern chatbots are a striking example of this engineering approach. They are built using neural networks to predict sequences of word-embedding vectors, the most recent key ingredient being the use of attention mechanisms, which enable sequential behaviour to be modelled using parallel computation (Vaswani et al., 2017). Subsequent models, including GPT-3 (Brown et al., 2020), Llama (Touvron et al., 2023), and DeepSeek (Liu et al., 2024), are trained and optimized to whole new levels of performance, though the core components of word embeddings, feed-forward neural networks, and attention heads using projection (with the whole system trained to optimize a loss function throughout the network using backpropagation) are fundamentally those established in the transformer model of Vaswani et al. (2017).

Like recursive and convolutional neural networks, transformer models use only word embeddings and word order (relative positions) to predict what words are likely to appear next (Widdows & Cohen, 2025, Chapter 4). This has been criticized as insufficient for intelligence, though it clearly coincides with Leibniz’ characterization of some of human intelligence:

In so far as the concatenation of their perceptions is due to the principle of memory alone, men act like the lower animals … Indeed, in three-fourths of our actions we are nothing but empirics. (Leibniz, Monadology, §28).

These models use none of the syntactic parsers, logical forms, or knowledge representation techniques that dominated much of late-1900s AI. Instead, they work much as (Turing, 1950) suggested: a ‘child machine’ is initialized with enormous computational resources and relatively few built-in procedures, just enough to learn from masses of training examples, after which it performs well—so well that its output, as exemplified by modern chatbots, produces language successfully enough that it would be considered a clear sign of intelligence in many established tests.

## 6. History of (Artificial) Intelligence Tests and Moving the Goalposts

Some of the early approaches attempted to test intelligence in terms of sensory ability (Galton, 1883; Jensen, 2011), an idea which was criticized by Wilhelm Wundt, who favoured complex cognitive thought, such as problem solving (Blumenthal, 1975; Wundt, 1896). Wundt’s ideas influenced the first standardised intelligence test, the Binet-Simon scale, and the idea of mental age, as a way to characterise developmental delays in intelligence (Binet & Simon, 1907).

The Binet-Simon scale inspired later intelligence tests, such as the Wechsler Adult Intelligence Scale (WAIS; Wechsler, 1939) and, in its US reincarnation (Terman, 1916), the Army Alpha (verbal-based) and Beta (non-verbal, for e.g., non-English speakers) tests, originally used for screening US Army recruits during the First World War (Yerkes, 1921). This work also led to the term intelligence quotient (IQ), the ratio of mental to chronological age (Stern, 1912). At around that time, researchers started recognising how some of these tests are culturally biased, favouring those from Western, educated, industrialised backgrounds (Bond, 1934). Such concerns led to the development of ostensibly culturally fair and non-verbal tests, such as Raven’s Progressive Matrices (Raven, 1938) and the culture-fair intelligence test (Cattell, 1943), an effort which continues to this date (Nisbett et al., 2012). An interesting assumption in these proposals is that verbal ability is largely due to education and so should not be part of intelligence testing. Today, successors to the Stanford-Binet test (Terman, 1916) and the WAIS test are still widely used, e.g., in clinical diagnosis, education, and cognitive functioning evaluations. Pertinently, there is evidence that even previous-generation chatbots (such as GPT-4) can solve a variety of problems used in IQ tests, including mathematics, verbal reasoning, comprehension of complex ideas, etc. (Bubeck et al., 2023), as well as perform highly on standard college applicant aptitude tests, such as the Stanford Achievement Test (SAT) and the Graduate Record Examination (GRE; OpenAI, 2023). Strong performance has already been observed with the type of problems typical in Raven’s Progressive Matrices test (Webb et al., 2023) and the recent versions of the WAIS. In February 2025, Grok AI announced expert-level achievements on advanced math (AIME), science (GPQA), and programming (LiveCodeBench) tests (xAI Team, 2025). While some chatbots (such as GPT-4) do not perform as well, this can often be traced to technical limitations of such models (e.g., visual processing; Hernández-Orallo, 2017).

Using tests designed for human examinees to benchmark AI systems has attracted recent attention, enabled by the sophistication of today’s AI models. In previous decades, AI testing methodologies have typically focused on basic competencies, such as the proportion of working transistors. Software tests have traditionally been carried out at different levels: unit testing checks that individual functions perform their calculations correctly; integration testing checks that the parts, when assembled, demonstrate the expected overall system behaviours; and regression testing checks that an updated version or specific instance of a system does not unexpectedly fall behind established performance expectations. None of these processes has been called ‘intelligence testing’, though there are analogies with the approaches of Binet and Simon (1907) and educational testing since, for example, the 2 times table is such a simple test, it should be passed by all students and assumed to be known thereafter.

Evaluation in machine learning has gradually adopted a more statistical approach, as outlined in Section 5. Instead of asking human experts to engineer rules for how to classify objects as a basis for (initially) relevance judgments (Cleverdon et al., 1966) and (later on) AI, human experts were asked to judge what the results should be. These judgements or annotations are then used as training examples for assessing AI accuracy. Examples include relevance judgments for document retrieval, what characters should be recognized in a handwriting sample, and whether an email message is spam or junk mail (Cormack, 2008). The use of standardized measures on particular datasets raises important questions, including sampling bias and whether a good benchmark result guarantees appropriate behaviour in real applications. Another challenge is that human judges do not always agree: measuring and studying inter-annotator agreement, or inter-rater reliability, is an important part of the methodology (Hallgren, 2012), though once an annotated dataset is available, it is common for machine learning evaluations to ignore this issue, even referring to labels from human annotators as ‘ground truth’. Notwithstanding these issues, the gradual adoption of established datasets and quantitative evaluations is part of what has enabled AI to make consistent progress since the 1990s, including the use of BLEU scores to evaluate machine translation (Bahdanau et al., 2014; Vaswani et al., 2017), and the development of comprehensive sets of language processing benchmarks designed to cover a variety of competencies (Wang et al., 2018).

Evaluation of systems by competing directly with human performance has been less of a motivating force in AI until recently. Initially, it was obvious that computers were faster and more reliable at arithmetic calculations (this is what they were built for), so there was little point in comparing human with mechanical performance, just as we would not race a powered vehicle against a human runner. By contrast, most other human behaviours regarded as intelligent were clearly beyond early computers. Games like chess and Go became exceptions, but organized competitions for Turing-like tests (particularly the prize offering by AI inventor Hugh Loebner) have declined since the late 2010s (Singh & Beniwal, 2022) (though just as this paper was being written, the experiments of Jones & Bergen, 2026, found that GPT-4.5 fooled human judges into choosing it as the ‘real’ human in an astonishing 73% of cases). These results can be taken to show that the Turing test, at least as initially conceptualised, can now be considered passed, and in recent discussions this is the assumption (e.g., E. K. Chen et al., 2026). Note that, while the Turing test has had particular historical importance in this debate, there are questions about its validity (Laddaga & Robertson, 2025), and it should be considered only one piece of evidence amongst many, regarding the question of chatbot intelligence.

Overall, the recent strong performance of AI systems on traditional intelligence tests for humans is not the culmination of decades in pursuit of this goal: it is more the result of decades of progress in lower- level benchmark evaluations, with the surprising outcome that systems trained to predict next words in a document or appropriate answers to relatively simple questions ended up performing well on tests designed for humans.

By the early 2010s, it was already clear that natural language processing systems could reach human-level performance on some language tasks, including syntactic tagging, information extraction, and textual inference. This motivated the development of new tests designed to highlight areas that are easy for humans and where AI was demonstrably still weak. For example, the Winograd Schema Challenge (named after a pioneering advocate for the study of ‘common sense’ in AI) asked simple questions like:

The trophy doesn’t fit in the brown suitcase because it’s too big. What is too big?

Answer 0: the trophy. Answer 1: the suitcase. (Levesque et al., 2012).

As emphasized by Chollet (2019), a pioneer of more general tests in AI, challenges like this are designed to stimulate, not to disparage, progress in AI, and sure enough, several systems had successfully tackled the Winograd Schema Challenge by 2020 (Kocijan et al., 2023). Chollet (2019) proposed a set of challenges called the Abstract Reasoning Corpus (ARC), which focuses on shapes in grid patterns and how they might interact physically: for example, it is easy for humans to see that a red shape is moved until it collides with a blue shape and to apply the same ‘rule’ immediately to the next scene. As of early 2025, performance of AI systems on ARC tests has leaped dramatically and, while there is discussion on whether the test has been conclusively passed (due particularly to energy considerations—the official ARC test included energy-consumption limits that were breached by GPT-03), Chollet (2024) praises the groundbreaking progress in AI.

**Figure 2:**
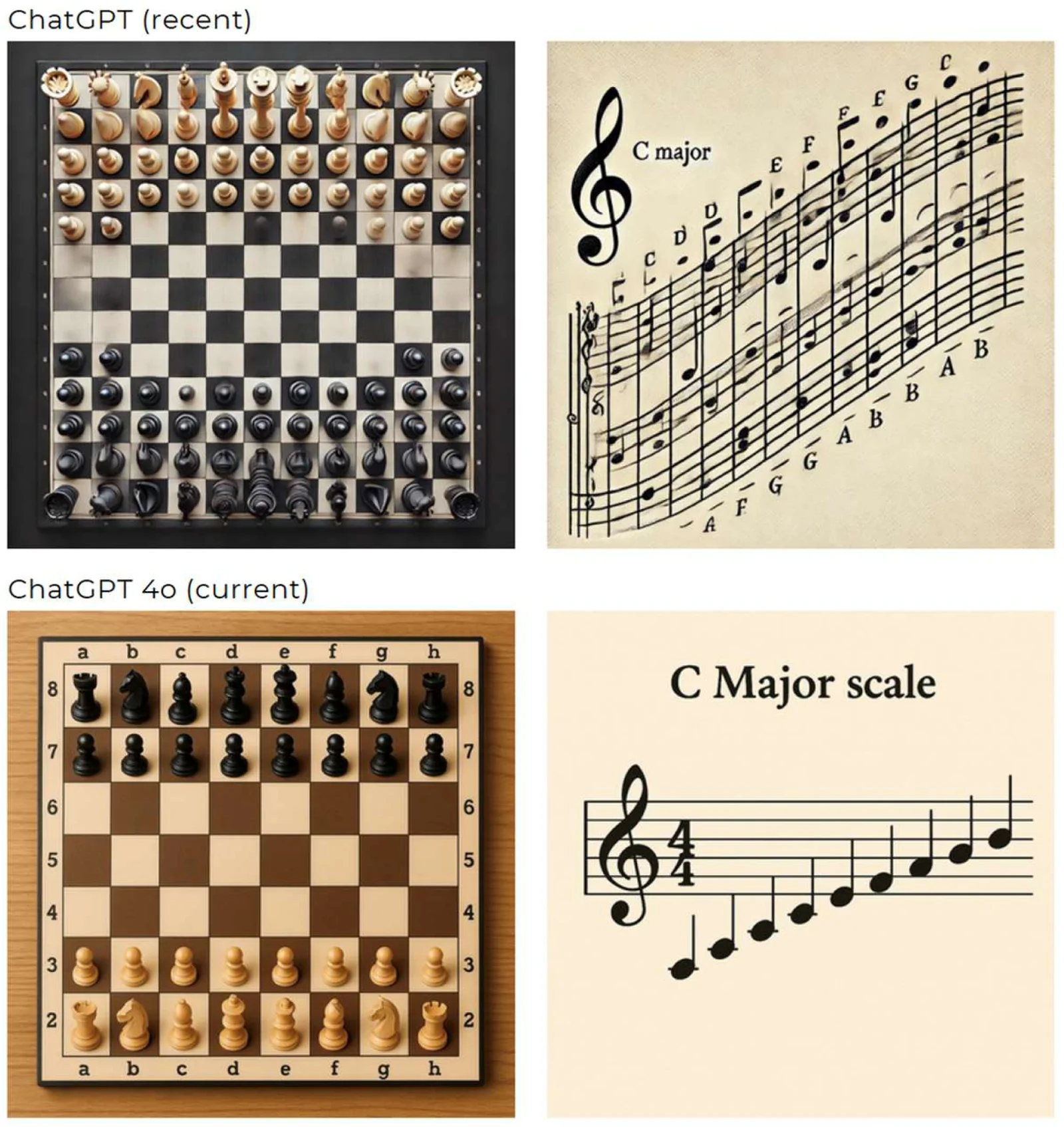
Examples of image generation, showing artistic flair but lacking accuracy. Images generated using ChatGPT, 1 March 2025 (above), and 7 April 2025 (below, after 4o upgrade). The prompt for the chessboards was “Please draw a diagram of a chessboard at the beginning of the game.” Regarding the music scores, the prompt was “Please draw a picture of a C major scale ascending from middle C in musical notation.”

It is easy to find evidence that connecting words with images in an exact fashion is still challenging for AI systems. State-of-the-art AI systems can generate stylish images from text prompts, but without preserving key facts: for example, a C major scale should have the notes CDEFGABC, and a chessboard at the beginning of the game should have exactly 64 squares and 32 pieces; until recently, AI-generated images used to fail to meet these simple requirements (Figure 2). Such gaps are perhaps even more surprising given the achievements of these models at tasks considered hard for humans (including the skilful drawing of shape, texture, and shading). Though note that more recent chatbot versions can produce outputs already better in diagrammatic accuracy. That is, even though perceptual processing appears to be a challenge for chatbots, there is good indication of improvement in leaps.

Overall, there is a long history of AI agents, including the most recent chatbots, trying to overcome challenges thought to be critical for showing (artificial) intelligence and aptitude tests designed for humans, even at an advanced level. Yet, it may like look that, as soon as an artificial system passes the current gold standard test for intelligence, we decide that the test was inadequate and invent a more elaborate one. That is, every time an AI test is passed, we move the goalposts and change our definition of intelligence, almost seemingly deliberately so as to keep out the most sophisticated AI system. A critical perspective would be that we just rig the game to retain a perception of superiority. Some such examples are noted above (e.g., chess), but there are others: having a flawless conversation with another human or translating a document into other languages would have been unquestioned signs of intelligence, until computers could do such tasks. But an alternative perspective is that revisions in AI tests also reflect an increasing understanding of what intelligence is. We think both perspectives have some truth to them. Indeed, even though currently it looks like chatbots pass all the tests we would consider relevant in assessing intelligence, we cannot preclude that, as our insight regarding intelligence further develops, we will be forced to revise current conclusions.

## 7. The Relation Between Embodiment and Intelligence

As we have seen, our approach to intelligence has been massively biased towards language and symbol-based tasks (such as mathematics): it is hard for us to imagine human civilisation without language and so it has been hard to imagine a notion of intelligence which is not founded on linguistic and symbol-manipulation abilities. However, the psychological (psychometric) literature on intelligence has recognised other types of intelligence too (Section 2). Regarding the chatbot debate, might we be missing something by ignoring embodiment?

Human intelligence is tied to the way we perceive and manipulate objects in the world (Shapiro & Spaulding, 2021), as well as our cultural and social context (Cobb, 2001). Some researchers go as far as to suggest that all knowledge we have is either embodied or has its origins in embodied knowledge (Lakoff & Johnson, 1999, 2003). For example, even in the most abstract scientific theories we have constructed, such as quantum mechanics, some of the basic ideas are extensions of embodied concepts (e.g., waves and particles); the most profound conceptual challenges in such theories come from ideas with no embodied analogues (e.g., superposition, non-locality). Moreover, our particular embodiment is tied to how we understand thought: many aspects of language reflect metaphorical extension of meaning from embodied concepts. For example, as soon as we say a problem is hard and takes a long time to solve, we demonstrate a spatial grounding to our concepts (Widdows, 2004, Chapter 1). But, as Gardner’s account of musical intelligence reminds us, not all thinking takes linguistic form (Gardner, 2011). In Phaedrus, Plato joins λόγος with rhythm, memory, and harmony as qualities of a well-ordered soul; learning “by heart” and reasoning through sound or motion are ways of knowing, and Plato places them closer to the psyche (ψυχή, often translated as soul) than the written word. These attributes vitally involve embodiment: so, the importance of embodiment is deeply established in Western philosophy but has become de-emphasized.

Notably, a great effort has gone into inventing ‘disembodied’ tests of intelligence, not tied to our specific embodiment (Section 6), at least in industrialized Western societies since the 1800s. This approach has led to an over-emphasis on intelligence tests based on language and manipulation of information through linguistic means (Section 4). Chatbots expose a fatal weakness to such tests, because, even though they are supremely capable wordsmiths, we are left uncertain as to whether they are intelligent or not and wondering if humans should be considered as genuinely inferior if chatbots beat us at, e.g., reasoning challenges.

Going back to embodiment, we wish to make two points. First, the kind of intelligence different agents develop must depend on embodiment; that is, we align with the strong embodiment thesis (e.g., Dreyfus, 1992; Varela et al., 1991; for a discussion specific to natural language processing see Bisk et al., 2020). We also think that a general notion of intelligence should be versatile enough to recognise different kinds of intelligence, arising from different embodiments. Regarding chatbots, they certainly have an indirect sense of the world, and it is a surprise that they talk as if they understand it, even when their experience is reduced to digitized text (Summerfield, 2024). Nevertheless, they mostly lack embodiment, and so their kind of intelligence would be expected to differ from ours.

Second, as current chatbots mostly lack embodiment, their embodied intelligence lags behind that of humans in most tasks. One could say that the key question for modern chatbots is less “Can chatbots think?” and more “Can chatbots see what they are doing?”—that is, the problem is not about the thought process, but about basic awareness. So, this is an aspect of intelligence in which humans are superior to chatbots. A related issue is that chatbots have limitations on contextual awareness. For example, chatbots sometimes propose syntactically-correct changes to code that have alarmingly bad consequences (Kim & Yegge, 2025). Note that some perceptual limitations are imposed by design: for example, a chatbot might be restricted from seeing menu options that elevate its own permissions. Still, limitations arising from embodiment are well-recognized and are being gradually addressed; for example, vision-aware models already include interfaces for sharing images (e.g., Lu et al., 2024). Moreover, advances in robotics will likely allow fully embodied chatbot agents before long (Anderson, 2003; H. Zhang et al., 2023). Robots that dance and perform typical human physical activities are already being marketed for consumer use. It is easy to anticipate machines that will win at tennis before long, in the same way that, by the early 1990s, it had become easy to anticipate that sooner or later machines would win at chess (Kasparov, 2017). One might counter-argue that the prototypical evolutionary example of human prowess in embodied and social intelligence might be reflected in the collaboration in a hunting or war party. However, it is hard to imagine that even the best human commandos would defeat a fleet of military drone hunters within a few years (Summerfield, 2024). Overall, increasingly, we are getting used to AI agents ‘seeing’. We are already used to voice tools like Siri and Alexa ‘hearing’.

Finally, the discussion of embodiment can help disentangle manual competencies from intelligence, a challenge which arises both for human and artificial intelligence (c.f., Aristotle’s Metaphysics, Bk 1, Ch 1, and Chollet, 2019). We are part of a culture that has assumed, for some centuries, that wordsmithing is intelligence, whereas manual dexterity is “mere” skill, and the use of physical force is sometimes disparaged as brutish. A cardinal example is the phrase “the pen is mightier than the sword”—an adage celebrated even on the walls of the Library of Congress (the quote is from an 1839 play by Edward Bulwer-Lytton, in the mouth of Cardinal Richelieu, First Minister of France from 1622 to 1642). If we remove the warlike associations and propose more blandly that “the pen is more useful than the knife”, then the claim is obviously not true in general and depends on the work to be done. Regarding intelligence, we think the critical factor is not whether the task is embodied or not, but rather the complexity of the task. After all, Kasparov (2017) pointed out that we have a long history of overestimating the complexity of intellectual tasks (such as remembering chess positions), based on the difficulty most of us experience with such tasks, compared with the ease with which we can make a cup of tea.

## 8. Concluding Comments

To recap, chatbots have fundamentally challenged our notion of intelligence. One reason is the role of (especially written) language in human intelligence and civilization. Language has been a key facilitator of progress in our species, by allowing inventions which persist beyond the individual, collaboration/role differentiation, and communities of knowledge (Fernbach & Sloman, 2018). Historically, human linguistic ability has been a pillar in our self-concept of intelligence and assumption of superiority—so, with the emergence of chatbots, an observer might say that we have to either declare chatbots intelligent or argue that being a wordsmith is no longer enough and change the rules for determining intelligence that we have insisted on for centuries.

On the initial question, “Are humans uniquely intelligent?” summarizing the apparent trends, in many IQ tests (and other tests related to intelligence), chatbots already outperform most humans, and it should not be long before this is the case for all such tests. GPT-4’s own estimate of when this will happen is 2030. We might just refrain from concluding that chatbots already demonstrate Artificial General Intelligence (AGI), because of their underperformance, relative to humans, in certain (particularly embodied) tasks. For example, even in 2025, common sense blunders are still prevalent. Relatedly, chatbots often produce so-called hallucinations (Ji et al., 2023). Turing (1950) described these as errors of conclusion for machines at the time, arguing that such instances did not rule out machine intelligence. Summerfield (2024) points out that confabulations is a better way to describe such issues and are better related to human psychology. So, while there is no doubt that chatbots are prone to hallucinations, there is disagreement regarding implications for intelligence. Some authors, such as Bessis et al. (2026), argue that hallucinations are no different from human memory errors (including false memories, e.g., Howe, 2013) or other cognitive biases. Note that, in recent work, there has been evidence that the latest chatbots are less prone to cognitive biases than humans (Bessis et al., 2026; Imannezhad et al., 2026).

This conclusion is disruptive to the scientific establishment. Turing predicted that “at the end of the century the use of words and general educated opinion will have altered so much that one will be able to speak of machines thinking without expecting to be contradicted” (Turing, 1950). Turing’s timeline estimate turns out to have been optimistic, but a quarter of the way through the next century, we do have models that use various “thinking modes”, and we are used to messages saying “Thought for X seconds”. The processes themselves are partly inspired by human approaches to problem solving, for example, by breaking reasoning down into steps and checking each step (Wei et al., 2022). Following technology history, we can expect that a chatbot thinking ‘something’ will be as commonplace as a book saying ‘something’. The objection that it is not really thinking will remain active (cf. Searle, 1980, and related works), and this concern remains important: the situation is similar to Plato’s concerns in the Phaedrus dialogue, which urge the readers themselves to remember that reading something is no substitute for truly knowing something.

There are several objections to a conclusion of chatbot intelligence. First, one criticism is that AI systems can accomplish apparent intelligence with vastly more resources compared to human brains. For example, GPT-4 has approximately one trillion parameters and an estimated cost of about $100 M to train, while Deep Blue had about 14,000 specialized chess chips. However, comparing complexity between human and artificial agents is not straightforward. The human brain is thought to have more than 80 billion neurons and 100 trillion neuronal connections (Azevedo et al., 2009; Herculano-Houzel, 2012), leading some scientists to claim that it is “…the most complicated object in the known universe” (the quote is attributed to physicist Kaku). Moreover, the brain has optimised many hardwired structures, making it efficient in cognitive tasks related to our physical constraints, environment, and needs. This optimisation has taken place across hundreds of millions of years for some generic abilities (e.g., visual perception; the time references the emergence of the first mammals, Kemp, 2005) and across hundreds of thousands of years for cognitive abilities more specific to humans (the time references the appearance of Homo Sapiens, Hublin et al., 2017; see also Section 3). So, while it might appear that some of these AI systems have frightening complexity, our brains are not exactly simple either. One might counterargue that the human brain still appears vastly more efficient than artificial systems, e.g., regarding most everyday learning tasks (including learning language). However, as just noted, the issue of efficiency of artificial vs. human agents, in the case of humans, needs to take into account both individual and evolutionary intelligence (this is a challenge which has yet to be addressed).

Second, another possible objection is that some of the AI solutions to particular problems may appear uncompelling, because they involve brute force approaches. For example, Deep Blue can evaluate approximately 200 million positions per second and, even though it uses various pruning algorithms to reduce calculation, it still searches through millions of moves (Campbell et al., 2002). As noted, Chollet’s (2019) ARC test, intended to be the new gold standard regarding testing for AI, has recently been partially overcome by GPT-o3 Chollet (2024). Recent developments leading to this achievement include Chain-of-Thought prompting, which automates the process of breaking a problem into basic steps (Wei et al., 2022), and test-time scaling, which devotes extra computational resources at inference time to check and revise answers (Muennighoff et al., 2025). The techniques used by such systems could be described as successful implementations of the proverbial advice “think before you speak”. Arguably, the success of inference-time improvements is just the application of brute force computation outside the training process. However, the human brain is also an example of a brute force approach to intelligence, if not in how we solve problems, certainly in how evolution has optimised cognitive processes for the range of problems relevant to us (as argued just above). We suggest that the reluctance to call, e.g., Deep Blue intelligent is less so about its brute force approach and more about domain specificity: we are unlikely to call intelligent an agent who excels (however impressively) in a single domain. A good example here is that of a pocket calculator, which excels in a particular domain of ’cognition’, yet is woefully inadequate in everything else. We think specificity is one key distinguishing characteristic of just skill vs. intelligence (cf. Chollet, 2019; Gignac & Szodorai, 2024).

Third, as a reviewer put it, “Just because chatbots pass IQ tests doesn’t mean these tests measure what we think they measure…when AI succeeds on tests designed to require “common sense,” we learn either that the tests don’t require what we thought, or that AI has developed that capacity.” A counterargument is to ask why it matters how an agent solves a test. Indeed, with humans, the same score on a test could be the result of widely different approaches, strategies, etc. Moreover, it is hard to imagine how the issue of ‘how’ can be resolved in a general way. Rather, the emphasis should be on having intelligence tests of sufficient complexity, so that the ‘how’ issue is less pertinent.

Fourth, there are some subtle issues regarding self-monitoring/self-awareness and intelligence. With chatbots, there is a degree of process monitoring not possible with humans: pulling open neural networks and language models to find how best to improve them is always possible, which gives us options for improvement that we can explore systematically. This advantage is alluded to already in LeCun et al. (1989) with the paper title “Optimal Brain Damage.” Regarding whether an AI system can be self-aware like a human is one of the objections considered by Turing (1950), who regards it as an appeal to solipsism (self -awareness is a personal experience, hence not something an individual can claim on behalf of a species). The issue of self-monitoring is easier to resolve: when asked about a conversation, chatbots are increasingly able to explain why they responded in a particular way (Li et al., 2025) and can sometimes recommend improvements in their own configuration to avoid problems recurring. For example, they can suggest updates to AGENTS.md files, which are used by developers to control coding agents.

Does self-awareness over and above self-monitoring matter for intelligence? One could propose that, from an engineering point of view, we should only care about the latter, since if an agent can monitor and improve its own behaviour, this is the only capacity that matters to its collaborators. However, the functional role of self-awareness in intelligence, if any, is currently unclear. Regarding current chatbots, while they show a fair amount of capability regarding self-monitoring, as far as we know, they are not currently self-aware (see also Gignac, 2026).

Fifth, and related to the above issues, there is the question of ‘understanding’. Searle’s Chinese Room (Searle, 1980) contends that syntactic manipulation of symbols, however fluent, does not entail semantic understanding; Harnad’s Symbol Grounding Problem (Harnad, 1990) makes the related claim that symbols derive meaning only through non-symbolic, sensorimotor connection to the world they represent; and Bender et al.’s “stochastic parrots” critique (Bender et al., 2021) argues that LLMs’ statistical mastery of linguistic form is being routinely and inappropriately conflated with genuine communicative intent or understanding.

Our position is as follows: each of these views presupposes that there is some further fact—“genuine” understanding—over and above the entire, extensive pattern of behaviour we would otherwise take as evidence for it: fluent, contextually appropriate, and generalisable engagement with the concept in question. But it is unclear what would count as evidence for this further fact, or against it, that is not already captured by the behaviour itself. For example, if a chatbot can discuss a house’s function, structure, materials, history, and cultural significance, draw analogies to other dwellings, correct misconceptions about it, and apply the concept flexibly to novel cases, the claim that it nonetheless lacks “true” understanding of what a house is becomes difficult to justify, on the basis of lacking some (additional) quality reflected in human thought. Put simply, if it walks like a duck and quacks like a duck, the burden falls on the sceptic to specify what additional, observable criterion is being withheld. Searle, Harnad, and Bender et al. are each, in their own way, raising exactly this question, but none resolve it decisively, and forty-five years after Searle’s original argument this remains a live philosophical dispute, rather than a settled matter. We do acknowledge that some readers will disagree with this position (e.g., Mahowald et al., 2024) and withhold a conclusion that chatbots are intelligent on such grounds.

Sixth, regarding chatbot performance on standardised tests, a well-documented objection relates to training data contamination, that is, the presence of benchmark items or close paraphrases in a chatbot’s training corpus (e.g., Jacovi et al., 2023). This is certainly an important issue. At the same time, chatbots also overcome new tests, including ones specifically designed to challenge them (such as in Chollet, 2019, 2024) and other tests typically tied to intelligence (such as university exams, Stribling et al., 2024; see also Gultom et al., 2024; Lehr et al., 2024). Noting that this is an extremely fast-moving field, converging evidence across several different sources, including standardized tests, tailor-made tests, and general competencies associated with intelligence (such as university coursework and exams), helps partly address the construct-validity concern. We offer the same response to the argument that chatbots are just able to ‘reverse engineer’ good performance on any specific test, without necessarily having the fundamental competency the test was designed to measure in humans.

In closing, we think that chatbots are close to Artificial General Intelligence and as intelligent as humans (if not more) on a range of competencies, which we have treasured as distinctly human for a long time. It increasingly appears that chatbots could replace humans in any task which is reduced to wordsmithing, symbol manipulation, etc., and the simple, but somewhat embarrassing, truth is that unique human competencies may correspond to the animal stuff—tasks in which we excel because of our particular embodiment, and for which a human empathic connection is useful. However, given the rapid development of multimodal AI models and semi-autonomous robots, this assessment may become dated within a few years. This is not necessarily a bad readjustment: we should perhaps start discarding anthropocentric biases in intelligence and start accepting that humans should not constitute a general standard of intelligence for all tasks. Even by 1950, Turing observed that humans no longer had any hope of performing arithmetic as fast as machines: so if arithmetic is one of the cognitive tasks attached to intelligence, humans cannot hope to surpass the ‘general intelligence’ of machines. Key advances in the scientific understanding of space and time have come from realising that man is not the measure of all things and human scales are small compared to the universe. Likewise, truly general intelligence need not be limited to the intelligence of a human. We hope that distinguishing these ideas will eventually help us to understand both intelligence and ourselves better.

## Data Availability

No new data were created or analyzed in this study. Data sharing is not applicable to this article.
